# Changes in endogenous cytokines, adhesion molecules and platelets during cytokine-induced tumour necrosis.

**DOI:** 10.1038/bjc.1995.481

**Published:** 1995-11

**Authors:** S. de Kossodo, R. Moore, S. Gschmeissner, N. East, C. Upton, F. R. Balkwill

**Affiliations:** Biological Therapies Laboratory, Imperial Cancer Research Fund, London, UK.

## Abstract

**Images:**


British Journal of Cancer (1995) 72, 1165-1172

© 1995 Stockton Press All rights reserved 0007-0920/95 $12.00

### Changes in endogenous cytokines, adhesion molecules and platelets during cytokine-induced tumour necrosis

S de Kossodo[1], R Moore[1], S Gschmeissner[2], N East[3], C Upton[2] and FR Balkwill[1]

[1]*Biological Therapies Laboratory and* [2]*Electron Microscopy Unit, Imperial Cancer Research Fund, 44 Lincoln's Inn Fields, London WC2A 3PX, UK.* [3]*Clare Hall Laboratories, Imperial Cancer Research Fund, Blanche Lane, South Mimms, Potters Bar, Herts EN6 3LD, UK.*

**Summary** The aim of this study was to investigate mechanisms of anti-tumour activity and necrosis induced by combinations of tumour necrosis factor alpha (TNF-α) and interferon gamma (IFN-γ). In a breast cancer xenograft model, locally injected recombinant human TNF-α arrested growth of established tumours in the absence of overt necrosis. Macroscopic necrosis occurred when rat IFN-γ, which had no anti-tumour activity as a single agent, was given systemically. Treatment with TNF-α and IFN-γ caused focal engorgement of tumour capillaries with erythrocytes, intravascular recruitment of polymorphonuclear cells and platelet adherence to the tumour vascular endothelium 4 h after the combined treatment. This was followed by destruction of tumour vascular endothelium and both necrosis and apoptosis of tumour cells. Concomitant with these changes, semiquantitative reverse transcriptase–polymerase chain reaction (RT–PCR) analysis revealed the increase of stromal (murine) mRNA levels for TNF-α, TNF receptor 55 kDa, TNF receptor 75 kDa, intracellular adhesion molecule 1, vascular cell adhesion molecule 1, P-selectin and interleukin 6 (IL-6). Thus, the effect of the combined TNF-α and IFN-γ therapy involved the selective destruction of the tumour vasculature, death of tumour cells and increased expression of a series of stromal cytokines, cytokine receptors and adhesion molecules, which could be implicated in the observed events.

**Keywords:** tumour necrosis factor alpha; interferon gamma; human xenograft; platelets; cytokines

The concept of inducing haemorrhagic necrosis to treat tumours was first investigated systematically by Coley (1893). However, it was not until 1975 that a tumour-necrosing activity in the sera of BCG-primed endotoxin treated mice was isolated (Carswell *et al.*, 1975). Since then, two molecules responsible for this activity, TNF-α (cachectin) and TNF-β (lymphotoxin), have been purified, cloned, and produced in large amounts by recombinant technology (reviewed in Beutler, 1992). Their availability allowed extensive studies, both in animal models and clinical trials. Although initially TNF-α was hailed as a potent anti-tumour treatment, it later proved to induce only modest tumour responses accompanied by unacceptable side-effects (Feinberg *et al.*, 1988). Moreover, endogenous TNF-α has also been implicated in tumour progression, particularly in ovarian cancer (Naylor *et al.*, 1993).

Potentiation of the anti-tumour actions of TNF-α was obtained with IFN-γ in a variety of murine tumours and human tumour xenografts (Brouckaert *et al.*, 1986; Fransen *et al.*, 1986; Balkwill *et al.*, 1987). Later, Thom *et al.* (1992) reported improvement of the anti-tumour effect and higher cure rate of MCA sarcomas treated with paralesional administration of TNF-α, together with IFN-γ. In recent clinical experiments, high doses of locoregional TNF-α, combined with systemic IFN-α and local chemotherapy, mediated specific destruction of tumour vasculature, and complete tumour regression, in a majority of patients with melanoma, squamous cell carcinoma and soft tissue sarcoma, whose tumours are accessible to isolated limb perfusion (ILP) (Liénard *et al.*, 1992; Eggermont *et al.*, 1993). The impressive anti-tumour effects of this treatment seem to be associated with vascular-mediated damage (Renard *et al.*, 1994). This treatment however remains controversial: other groups using the same procotol have been unable to obtain similar results (Vaglini *et al.*, 1994).

These studies led us to develop an animal model reproducing some of the observed effects of TNF-α and IFN-γ in ILP therapy. There are obviously differences between ILP with human vascular administration of human TNF-α and human IFN-γ in a human host *vs* intratumoural injections of human TNF-α and systemic administration of rat IFN-γ in an immunocompromised mouse. However, we used a xenogeneic system to permit dissociation between effects on host and tumour cells. We examined the actions of TNF-α and IFN-γ on tumour necrosis, tumour growth and survival. Histological changes were monitored and expression of stromal cytokine, cytokine receptor and adhesion molecule mRNA were followed within the murine host.

Correspondence: S de Kossodo
Received 27 February 1995; revised 25 May 1995; accepted 13 June 1995

### Materials and methods

### *Mice*

Female pathogen-free *nu/nu* mice of mixed genetic background were bred by the Imperial Cancer Research Fund animal breeding unit, Clare Hall, Potters Bar, UK. They were housed in negative pressure isolators and used for experiments when 6–9 weeks old.

### *Tumours*

The human breast mucoid carcinoma 1068 was originally derived from a primary tumour (Balkwill *et al.*, 1986) and maintained by passage in mice at 6–8 week intervals.

### *Cytokines*

Recombinant human TNF-α kindly provided by BASF/Knoll (Maidenhead, Berkshire, UK), was more than 99% pure and contained less than 0.125 EU mg$^{-1}$ endotoxin. The sp. act. was $5 \times 10^7$ units mg$^{-1}$. Recombinant rat IFN-γ was provided by Roussel UCLAF (Romainville, France). It had a sp. act. of $1 \times 10^7$ units mg$^{-1}$. Endotoxin levels were less than 0.367 EU mg$^{-1}$. TNF-α and IFN-γ were diluted to the appropriate concentration with calcium- and magnesium-free phosphate-buffered saline (PBS) containing 3 mg ml$^{-1}$ bovine serum albumin (BSA; Sigma, Dorset, UK) and stored in single dose aliquots at −70°C.

*Experimental design*

Established tumours were minced finely with scissors and 0.05 ml tumour suspension was injected subcutaneously (s.c.) into a lateral site on each mouse. After 7–21 days when tumours measured approximately 0.2–0.4 cm, therapy was started. Each experimental group contained 7–8 mice bearing a single tumour. Mice were given daily or weekly intratumoural (i.t.) injections of 0.1 ml of the appropriate TNF-α dose or PBS/BSA control solution. Rat IFN-γ (0.1 ml) at the appropriate dose was injected daily or once s.c. near the site of the tumour. Tumours were observed daily for signs of necrosis and measured once weekly with calipers. The tumour size indices shown in the figures are a multiplication of the two largest tumour diameters at right angles to each other.

*Histology*

Tumour samples were fixed in buffered formalin and embedded in paraffin. Sections were cut at 5 μm and stained with haematoxylin and eosin. In certain experiments, samples were fixed in 2.5% glutaraldehyde in Sorensen's phosphate buffer and processed for electron microscopy using standard procedures. One micron toluidine blue sections were used to select representative areas for ultrastructural investigation.

*RNA preparation and cDNA synthesis*

RNA was isolated by a single extraction with guanidinium thiocyanate phenol chloroform mixture as described (Chomczynski and Sacchi, 1987). Total RNA (5 μg) was incubated for 30 min at 37°C in 40 mM Tris-HCl pH 7.5, 10 mM sodium chloride, 6 mM magnesium chloride and 2.5 units of RQ1 DNAse (Promega, USA) in order to remove any contaminating genomic DNA from the preparations. After phenol chloroform extraction and ethanol precipitation, pellets were resuspended in water. Synthesis of the first strand of cDNA was performed according to the instructions delivered with the cDNA Synthesis Kit (Boehringer Mannheim, Switzerland), using random primers and avian myeloid leukaemia virus (AMV) reverse transcriptase (10 units per sample). After 1 h incubation at 42°C, samples were diluted with water to a total volume of 50 μl, heat inactivated and kept frozen (−20°C) until use.

*Polymerase chain reaction analysis*

Aliquots of 2 μl of cDNA (the equivalent of 200 ng of total RNA) were amplified in a final volume of 25 μl using the Gene-Amp kit (Perkin Elmer, Norwalk, USA), in the presence of 5 μCi of [α-$^{32}$P]dCTP (3000 Ci mmol$^{-1}$, Amersham) and 2.5 units of ampli*Taq* (Perkin Elmer Cetus). Samples were overlaid with mineral oil and amplified at 94°C for 5 min, 60°C for 1 min and 72°C for 30 s followed by 35 cycles at 94°C for 30 s, 60°C for 1 min and 72°C for 30 s. The termination cycle consisted of one cycle at 94°C for 30 s, 60°C for 1 min and 72°C for 10 min. PCR was carried out in an automated DNA Thermal Cycler (Techne, Cambridge, UK), in the presence of 0.2μM of each primer. The following primers were used:

GAPDH 1, 5'-TGA AGG TCG GTG TGA ACG GAT TTG G-3'
GADPH 2, 5'-ACG ACA TAC TCA GCA CCA GCA TCA C-3'
TNF-α 1, 5'-CCC GAC TAC GTG CTC CTC-3'
TNF-α 2, 5'-GAC CTG CCC GGA CTC CGC-3'
TNF receptor 55 kDa 1, 5'-CCG GGC CAC CTG GTC CG-3'
TNF receptor 55 kDa 2, 5'-CAA GTA GGT TCC TTT GTG-3'
TNF receptor 75 kDa 1, 5'-GAC GAA TTC ATG GAG TAG GCC TTG AGC-3'
TNF receptor 75 kDa 2, 5'-TAA GGA TCC CTG AGA CGG ACA CTC CTC-3'
ICAM-1 1 5' CAA CTG GAA GCT GTT TGA GCT G-3'
ICAM-1 2 5'-TAG CTG GAA GAT CGA AAG TCC G-3'
VCAM-1 1 5'-CAA GGG TGA CCA GCT CAT GA-3'
VCAM-1 2 5'-TGT GCA GCC ACC TGA GAT CC-3'
P-Selectin 1 5'-ACG AGC TGG ACG GAC CCG-3'
P-Selectin 2 5'-GGC TGG CAC TCA AAT TTA CAG C-3'

The glyceraldehyde phosphatase dehydrogenase (GAPDH) primers recognise both human and murine GAPDH, the remaining primers are all based on published murine sequences. IL-6 primers were purchased from Clontech (Clontech Laboratories, Palo Alto, CA, USA). One-half of the reaction was electrophoresed on 1% agarose gels and the appropriate bands revealed by ethidium bromide staining of the gel.

Relative quantitation was achieved by subjecting parallel samples to amplification in subsaturating conditions of the constitutively expressed GAPDH gene and the specific cytokine, receptor or adhesion molecule. The relative product amounts were quantitated by measuring the incorporated radioactivity of the specific bands from dried gels with a Molecular Dynamics 400A PhosphorImager. The values obtained for each cytokine were normalised to the values obtained by measuring the incorporated radioactivity in the corresponding GAPDH amplification products. Under the conditions used there was a linear relationship between the initial amount of RNA and the PCR signal. It should be emphasised however that this protocol does not allow for a precise determination of the exact amount of mRNA molecules present in each sample.

*Statistical analysis*

The data were analysed for statistical significance between groups using the Student's *t*-test; (two-tailed). Differences in survival times were calculated by ANOVA.

### Results

*Effect of TNF-α and IFN-γ on necrosis, tumour growth and survival*

We first measured the effects of varying doses of human TNF-α and rat IFN-γ on macroscopic necrosis, tumour growth and treatment-related mortality (Table I). Only mice receiving IFN-γ in combination with TNF-α showed overt haemorrhagic tumour necrosis which appeared approximately 2 weeks after beginning the treatment. Mice injected with the highest TNF-α and IFN-γ doses (5 μg TNF-α and 50 × 10$^3$ U IFN-γ) developed generalised vascular collapse and tumour haemorrhagic necrosis so severe as to cause ulceration of the overlying epidermis. Less severe tumour necrotic patches, which generally resolved with scar formation, are observed in mice injected with 2.5 μg TNF-α + 5 × 10$^3$ U IFN-γ.

Tumour growth was inhibited with daily injections of 2.5 μg TNF-α, ($P = 0.01$ at 3 weeks, as compared to control, Figure 1a). IFN-γ (5 × 10$^3$ U) alone had no effect. Potentia-

**Table I** Effect of TNF-α and IFN-γ on tumour growth, necrosis and treatment-related mortality

| *TNF-α i.t. (μg)* | *IFN-γ s.c. × 10$^3$ units* | *Effect on tumour growth* | *Macroscopic necrosis* | *Mortality* |
|---|---|---|---|---|
| 5 daily | 0 | Yes | no | no |
| 0 | 50 daily | No | no | no |
| 5 daily | 50 daily | Yes | yes (14)[a] | yes |
| 5 daily | 5 daily | Yes | no | no |
| 2.5 daily | 25 daily | Yes | no | no |
| 2.5 daily | 5 daily | Yes | yes (14, 17)[b] | no |
| 2.5 daily | 25 once | Yes | no | no |

Mice received daily cytokine treatments starting 14 days after tumour cell inoculation. [a]Day of appearance. [b]Days of appearance, two separate experiments.

tion of the anti-tumour growth effect was achieved with daily injections of 2.5 μg TNF-α in combination with $5 \times 10^3$ U IFN-γ (Figure 1a and b, $P = 0.003$ at 3 weeks, as compared with control; $P = 0.049$ at 4 weeks, as compared with TNF-α alone). As described above, this regimen was accompanied by the appearance of tumour necrosis. Survival of treated mice was prolonged as compared with controls (Figure 2). Some animals had to be killed because overt haemorrhagic necrosis developed, however, mice seemed otherwise healthy. Two-way ANOVA test showed the difference in survival times was highly significant ($P = 0.004$) as compared with controls.

Maximum inhibition of tumour growth in the absence of macroscopic necrosis was obtained with 2.5 μg TNF-α + $25 \times 10^3$ U IFN-γ ($P = 0.013$ after 2 weeks of therapy, $P < 0.0001$ after 3 weeks, as compared with control, Figure 1b). This regimen however, was not significantly different, in terms of growth-inhibitory effect, to the 2.5 μg TNF-α + $5 \times 10^3$ U IFN-γ regimen. One hundred per cent of animals receiving this regimen survived 42 days after beginning the therapy, that is 52 days after inoculation of the tumour cells ($P = 0.00004$ as compared with controls) at the time when the experiment was stopped (Figure 2). Total tumour regression was observed in two out of seven mice having received the 2.5 μg TNF-α + $25 \times 10^3$ U IFN-γ therapy, 18 weeks after tumour inoculation.

**Figure 1** Effect of combined administration of TNF-α and IFN-γ on growth of 1068 human breast carcinoma xenografts. (**a**) Effect of single and combined TNF-α and IFN-γ treatment. (**b**) Effect of IFN-γ dosage in combined treatment. Mice received daily i.t. injections of human recombinant TNF-α and s.c. injections of recombinant rat IFN-γ, starting 14 days after inoculation of the tumour cells. Each experimental group consisted of seven mice. Mean values from one representative experiment are shown. *Tumour size was significantly different from control value. Differences were not calculated thereafter because control animals had to be sacrificed at 2 weeks.

**Figure 2** Survival of mice receiving daily combined TNF-α and IFN-γ treatment. Each experimental group consisted of seven mice. One representative experiment is shown. Animals injected with 2.5 μg of TNF-α and 5000 U of IFN-γ had to be sacrificed at 37 days because they developed large haemorrhagic necrosis of the area surrounding the tumour.

In control mice light microscopy showed tumour cell nodules consisting of interdigitating cells surrounded by a stromal reaction containing macrophages, eosinophils, polymorphonuclear cells, lymphocytes and small blood vessels (Figure 3a). By contrast, tumours in mice treated with 2.5 μg TNF-α and $25 \times 10^3$ U IFN-γ showed complete regression after 35 days of therapy (Figure 3b). Only normal breast ducts and fat cells of mouse origin were present, indicating that tumour cells had not differentiated into breast tissue but had altogether disappeared. Indication of apoptosis and fibrinoid necrosis preceded these changes.

*Vascular changes*

By electron microscopy, numerous capillaries could be found in the host cell stromal reaction area but none in the tumour cell islands. Figure 4a shows part of a small capillary from a control tumour. At 4 h after injection with 2.5 μg TNF-α and $25 \times 10^3$ U IFN-γ there was focal engorgement of tumour capillaries with erythrocytes. Intraluminal accumulation of platelets and polymorphonuclear cells was also observed (Figure 4b). At 6 h after therapy vessels were filled with erythrocytes and thrombi, composed of degranulated platelets and fibrin, were present. Accumulation of platelets and their close association to endothelium was observed in

**Figure 3** Histological appearance of 1068 breast carcinoma. (**a**) Tumour nodules (T) in control mice (after 14 days at which time mice had to be sacrificed). (**b**) Normal murine breast ducts (D) and fat cells (F) with no evidence of tumour cells in mice having received 2.5 μg of TNF-α and $25 \times 10^3$ U of IFN-γ for 35 days. Haematoxylin and eosin (magnification × 65).

areas of vascular injury (Figure 4c). After 24 h tumour capillary endothelial cells had disappeared, although the basal lamina appeared to be intact (Figure 4d). Not all capillaries in treated tumours exhibited these changes; some capillaries showed no intraluminal erythrocyte accumulation and in others the basal lamina had disappeared. In general, the areas of vascular damage tended to be focal and associated with areas of tumour cell death. These changes were not observed in normal skin injected with TNF-α and IFN-γ nor in tumours treated with either agent alone. No inflammatory cell infiltrates were seen within the tumour mass in either control or cytokine-injected mice.

### *Apoptosis and necrosis*

As shown in Figure 5, the injection of 2.5 μg TNF-α and $25 \times 10^3$ U IFN-γ induced tumour cell death by two distinct mechanisms. Certain cell were clearly apoptotic with characteristic condensation of chromatin around the periphery of the nucleus, together with round, less densely staining

**Figure 4** Platelet aggregation and adhesion to vascular endothelium in an area of vascular injury. (**a**) Normal endothelial cell (E). (**b**) Platelet aggregation (P) and adherence to endothelium 4 h after injection of 1068 xenoagraft with 2.5 μg of TNF-α and $25 \times 10^3$ U of IFN-γ. (**c**) After 6 h, intraluminal thrombus (T) composed of degranulated platelets and fibrin in area of endothelial cell damage. (**d**) At 24 h, disappearance of endothelium, only the basal lamina remaining (B). Bar = 1 μm.

nucleolar fibrillar centres. Other cells appeared to be in the early stages of chromatin condensation of apoptosis. Secondary necrosis with a rather haphazard pattern of disintegration of the cytoplasm could also be found. Early and late stages of necrosis, without evidence of prior apoptosis also appeared. Finally, cell fragments were present which could be due to late stage necrosis, although the changes were far too advanced to determine whether there was any prior apoptosis.

*Cytokines, cytokine receptors and adhesion molecules*

Total RNA from tumour was extracted from two mice at each time point following injection with either 2.5 μg TNF-α, 25 × $10^3$ U IFN-γ or both. mRNA expression was analysed by RT–PCR, using murine-based primers, so as to study cytokine and adhesion molecule expression changes in the host. To calculate the relative fold induction of mRNA, PCR-generated bands were quantitated with a PhosphorImager and the values obtained were corrected by ratio to their corresponding GAPDH amplification products. Induction was then estimated as the fold change compared with baseline (control) values. Given the inherent variations due to the technique, only increases above 5-fold or higher were considered significant. Low levels of murine TNF-α mRNA were detected in BSA-injected control 1068 tumours (not shown). Following the injection of either cytokine alone or in combination, endogenous murine TNF-α mRNA expression rose rapidly. Maximal induction of TNF-α mRNA appeared between 30 min and 1 h after therapy (Figure 6). Both TNF-α receptors 55 kDa and 75 kDa were induced by all treatments, with highest mRNA expression at 3 h post-

**Figure 5** Necrosis and apoptosis of 1068 breast carcinoma cells *in vivo*. (**a**) Normal tumour cells. (**b**) Presence of early apoptotic cells (A), early stage of apoptosis (EA), secondary necrosis (SN), early stage necrosis (EN) and advanced necrosis (N) in tumours 24 h after injection with 2.5 μg of TNF-α and 25 × $10^3$ U IFN-γ. Bar = 1 μm.

**Figure 6** Effect of TNF-α and IFN-γ on (**a**) TNF-α (**b**) TNF R 55 kDa and (**c**) TNF R 75 kDa mRNA levels. Tumour RNA was extracted from two mice at different times following injection with 2.5 μg of TNF-α or 25 × $10^3$ U of IFN-γ or both. BSA-injected animals served as control. Parallel samples were amplified for TNF-α and GAPDH and the specific PCR-generated fragments visualised by ethidium bromide staining of the gel. To show the fold induction, TNF-α, TNF R 55 kDa- and TNF R 75 kDa-specific fragments were amplified by RT–PCR, in the presence of a radiolabelled nucleotide. Appropriate bands were quantitated with a PhosphorImager and the values obtained were corrected by ratio to GAPDH amplification products (upper right and below). Values shown are the mean of two mice per treatment per time point and represent the fold difference as compared with control values. □, IFN-γ; ■, TNF-α; ▨, TNF-α and IFN-γ.

**Figure 7** Effect of TNF-α and IFN-γ on IL-6, ICAM-1, VCAM-1 and P-selectin mRNA levels. Tumour RNA was extracted from two mice at different times following injection with 2.5 μg of TNF-α or 25 × $10^3$ U of IFN-γ or both. BSA-injected animals served as control. IL-6, ICAM-1, VCAM-1 and P-selectin mRNA expression was analysed by RT–PCR, in the presence of a radiolabelled nucleotide. Appropriate bands were quantitated with a PhosphorImager and the values obtained were corrected by ratio to GAPDH amplification products. Values shown are the mean of two mice per treatment per time point and represent the fold difference as compared with control values. □, IFN-γ; ■, TNF-α; ▨, TNF-α and IFN-γ.

therapy (Figure 6). Intercellular adhesion molecule-1 (ICAM-1) mRNA was also detected in control tumours and was strongly induced by either therapy, with maximum mRNA levels appearing 3 h after injection, returning to baseline levels after 24 h (Figure 7). Vascular cell adhesion molecule-1 (VCAM-1) mRNA levels were almost undetectable in control tumours. mRNA expression was induced by either cytokine alone or in combination, with highest VCAM-1 mRNA expression reached 6 h post therapy. P-selectin mRNA was present in low levels in control tumours and was also rapidly induced by either treatment. Maximum induction was achieved 1 h after therapy (Figure 7). IL-6 mRNA was very strongly induced by TNF-α treatment but not by IFN-γ after 1 h. Dual therapy did not affect significantly the expression of IL-6, as compared with TNF-α alone (Figure 7).

### Discussion

In the present study we have shown that high-dose human recombinant TNF-α, delivered locally, was able to inhibit tumour growth. Human TNF-α can only act through the 55 kDa receptor but not the 75 kDa receptor in mice (Brouckaert *et al.*, 1993). The TNF R 75 kDa has been shown to mediate many of the vascular effects of TNF-α, such as neutrophil adhesion to the endothelium and E-selectin expression (Barbara *et al.*, 1994). However, we have observed a very early induction of murine TNF-α mRNA (30 min) in response to human recombinant TNF-α or rat IFN-γ therapy, which in turn may account for specifically 75 kDa-mediated effects. Potentiation of the anti-tumour effect was achieved by combining TNF-α with systemically administered IFN-γ. The role of IFN-γ in the potentiation of the anti-tumour effects of TNF-α is critical and is well documented (Fransen *et al.*, 1986; Chen *et al.*, 1987). In agreement with our observations, Thom *et al.* (1992) have reported effective treatment of MCA sarcomas in mice with paralesional TNF-α and IFN-γ therapy, as well as local skin necrosis leading to treatment-related mortality. In patients, treatment of malignant melanomas with TNF-α, IFN-γ and melphalan by isolated limb perfusion has provided controversial results. On the one hand, investigators reported a high success rate in a majority of patients with melanoma, squamous cell carcinoma and soft tissue carcinoma (Liénard *et al.*, 1992, 1994; Eggermont *et al.*, 1993). However, other groups following the same protocol have not been able to obtain similar results (Vaglini *et al.*, 1994).

Tumour regression in response to TNF-α can be due to direct and indirect effects, many of which may be potentiated by IFN-γ. Among its direct effects TNF-α can induce both apoptotic and necrotic forms of cell lysis (Laster *et al.*, 1988; Bellomo *et al.*, 1992). In the present report, we have shown necrosis and apoptosis of 1068 tumour cells. It is not clear, however, whether necrosis was the direct result of TNF-α, or the indirect result of vascular damage, oxygen starvation, cytotoxic actions of other host stromal cells induced by the therapy or a combination of these. IFN-γ, which can also induce either pathological or physiological cell death (Aune and Pogue, 1989) could not have caused tumour cell death directly, since rat IFN-γ does not act on human cells.

Vascular damage is now widely accepted as a mechanism of the tumoricidal actions of TNF-α. This cytokine has been reported to have direct cytotoxic effects on the vascular endothelium (Watanabe *et al.*, 1988), to induce apoptosis of endothelial cells *in vitro* (Robaye *et al.*, 1991) and to modulate endothelial procoagulant properties (Nawroth and Stern, 1986). Thrombus formation and fibrin deposition are common histological findings in TNF-α-treated Meth A sarcomas (Shimomura *et al.*, 1988). It is also worth noting that anticoagulants have been shown to inhibit the anti-tumoural effects of TNF-α (Shimomura *et al.*, 1988). In Friend leukaemia tumours and fibrosarcomas injected peritumourally with TNF-α, there is evidence of vascular congestion, intraluminal thrombi and the accumulation of platelets, in association with marked damage of endothelial cells before the appearance of necrotic areas (Proietti *et al.*, 1988). Injury to endothelial cells and accumulation of neutrophils was also reported after TNF-α and hyperthermia therapy in the RIF-1 fibrosarcoma (Srinivasan *et al.*, 1990). Furthermore, Renard *et al.* (1994) have observed early endothelial cell activation and polymorphonuclear cell invasion preceding necrosis in human tumours treated with high-dose TNF-α and IFN-γ by ILP therapy. These histological descriptions correlate with our present observations of intravascular accumulation of erythrocytes and neutrophils, platelet adherence to the tumour vascular endothelium followed by endothelial cell damage and haemorrhagic necrosis.

The question arises as to the actual mechanisms of endothelial cell injury. Recent studies have provided evidence for an unexpected role for platelets in mediating vascular pathology. Grau *et al.* (1993) have demonstrated the pivotal role played by platelets in cerebral malaria. In susceptible animals, high circulating levels of TNF-α and increased local production of IFN-γ lead to the overexpression of ICAM-1 in brain microvascular endothelial cells. Leucocyte function antigen-1 (LFA-1)-bearing platelets then specifically adhere to ICAM-1 and fuse with endothelial cells, causing irreversible damage and microhaemorrhages. It is tempting to speculate that a similar sequence of events leads to endothelial destruction and haemorrhagic necrosis in the 1068 tumour following high-dose local TNF-α and IFN-γ therapy.

TNF-α and IFN-γ can promote the expression of overlapping sets of adhesion molecules (Pober *et al.*, 1986). Following cytokine therapy, we noted the increased expression of endogenous TNF, TNF receptor 55 kDa, TNF receptor 75 kDa, ICAM-1, VCAM-1, P-selectin and IL-6 mRNA levels. To the best of our knowledge, this is the first extensive study investigating the kinetics of mRNA expression for these cytokines, receptors and adhesion molecules in tumours. In general, low levels of ICAM-1 and VCAM-1 can be observed in normal vascular endothelium and their expression is stimulated by cytokine therapy (reviewed in Bevilacqua 1993). VCAM-1, as well as P-selectin, can also be present in non-vascular cells. Mediators that stimulate P-selectin expression can cause endothelial cell retraction or damage resulting in increased permeability. Furthermore, higher levels of ICAM-1 mRNA in response to TNF-α and IFN-γ therapy could be involved in the adherence of platelets to the tumour vascular endothelium and the ensuing damage, by analogy with the pathology observed in the murine model of cerebral malaria (Grau *et al.*, 1993). However, this alone cannot account for tumour regression since IFN-γ has no activity on its own. ICAM-1 can also bind LFA-1-expressing neutrophils and monocytes. When neutrophils adhere to activated endothelium they can secrete proteolytic enzymes which are able to destroy the extracellular membrane (Cajot *et al.*, 1986). The induction and amplification of adhesion molecule expression, namely VCAM-1, ICAM-1 and ELAM-1 have also been reported in melanoma and sarcoma patients treated with high dose TNF-α, IFN-γ and melphalan (Renard *et al.*, 1994). The specific induction of IL-6 by TNF-α alone or in combination with IFN-γ is of interest. After continuous infusion of TNF-α and IFN-γ, elevated circulating levels of IL-6 have been reported (Brouckaert *et al.*, 1989). In the study presented here, IL-6 could thus contribute to the anti-tumoural effect, as shown before in other murine tumour models (Mule *et al.*, 1990). Very recently, increased serum Il-6, ICAM-1 and TNF receptor 55 kDa concentrations have been detected in patients undergoing TNF-α/IFN-γ/melphalan therapy by ILP (Thom *et al.*, 1995).

In conclusion, the present study shows that we have been able to produce a useful model of cytokine-induced tumour growth arrest and necrosis, comparable with ILP (Liénard *et al.*, 1992). The differences between human ILP and the present model should however be stressed: in ILP, human TNF-α is injected systemically, in combination with human IFN-γ and melphalan. We inject human TNF-α intratumourally and IFN-γ systemically. In ILP, a human tumour in a human host is treated, whereas we treat a human xenograft in an immunocompromised mouse. Notwithstanding the limitations imposed by our model, we found it to reproduce certain key aspects of ILP: destruction of tumour cells by necrosis, endothelial cell activation, induction of adhesion molecule expression and specific damage of the tumour vasculature in response to TNF-α and IFN-γ therapy. As for ILP, it is tempting to speculate that the association of platelets and neutrophils with areas of vascular injury, via the induction of specific adhesion molecules on the host endothelium, may play a crucial role in mediating these anti-tumoural actions.

**Acknowledgements**
We wish to thank Dr Mark Arends for lending us his expertise in identifying necrotic and apoptotic events and Mr George Elia and the ICRF Histopathology Unit for histopathology support. We also thank BASF/Knoll and Roussel UCLAF for providing TNF-α and IFN-γ. We are grateful to the Fonds National Suisse de la Recherche Scientifique (Switzerland) for their support.

### References

AUNE TM AND POGUE SL. (1989). Inhibition of tumor cell growth by interferon-gamma is mediated by two distinct mechanisms dependent upon oxygen tension: Induction of tryptophan degradation and depletion of intracellular nicotinamide adenine dinucleotide. *J. Clin. Invest.*, **84**, 863–875.

BALKWILL FR, LEE A, ALDAM G, MOODIE E, THOMAS JA, TAVERNIER J AND FIERS W. (1986). Human tumour xenografts treated with recombinant human tumor necrosis factor alone or in combination with interferons. *Cancer Res.*, **46**, 3990–3993.

BALKWILL FR, WARD BG, MOODIE E AND FIERS W. (1987). Therapeutic potential of tumor necrosis factor-α and γ-interferon in experimental human ovarian cancer. *Cancer Res.*, **47**, 4755–4758.

BARBARA JA, SMITH WB, GAMBLE JR, VAN-OSTADE X, VANDENABEELE P, TAVERNIER J, FIERS W, VADAS MA AND LOPEZ AF. (1994). Dissociation of TNF-alpha cytotoxic and proinflammatory activities by p55 receptor- and p75 receptor-selective TNF-alpha mutants *EMBO J.*, **13**, 843–850.

BELLOMO G, PEROTTI M AND TADDEI F. (1992). Tumor necrosis factor-α induces apoptosis in mammary adenocarcinoma cells by an increase in intranuclear free $Ca^{2+}$ concentration and DNA fragmentation. *Cancer Res.*, **52**, 1342–1346.

BEUTLER B. (ed) (1992). *Tumor necrosis factors. The molecules and their emerging role in medicine*. Raven Press: New York.

BEVILACQUA MP. (1993). Endothelial-leukocyte adhesion molecules. *Ann. Rev. Immunol.*, **11**, 767–804.

BROUCKAERT PGG, LEROUX-ROELS GG, GUISEZ Y, TAVERNIER J AND FIERS W. (1986). *In vivo* anti-tumor activity of recombinant human and murine TNF, alone or in combination with murine IFN-gamma, on a syngeneic murine melanoma. *Int. J. Cancer*, **38**, 763–769.

BROUCKAERT P, SPRIGGS DR, DEMETRI G, KUFE DW AND FIERS W. (1989). Circulating interleukin 6 during a continuous infusion of tumor necrosis factor and interferon gamma. *J. Exp. Med.*, **169**, 2257–2262.

BROUCKAERT P, EVERAERDT B, LIBERT D, TAKAHASHI N, CAUWELS A AND FIERS A. (1993). Strategies to broaden the therapeutic margin of tumour necrosis factor. In *Tumor Necrosis Factor: Molecular and Cellular Biology and Clinical Relevance*. Fiers W and Buurman W (eds) pp. 226–232. Karger: Basle.

CAJOT JF, KRUITHOF EKO, SCHLEUNING WD, SORDAT B AND BACHMANN F. (1986). Plasminogen activators, plasminogen activator inhibitors and procoagulant analyzed in twenty human tumour cell lines. *Int. J. Cancer*, **48**, 719–727.

CARSWELL EA, OLD LJ, KASSEL RJ, GREEN S, FIORE N AND WILLIAMSON B. (1975). An endotoxin-induced serum factor that causes necrosis of tumours. *Proc. Natl Acad. Sci. USA*, **72**, 3666-3670.

CHEN L, SUZUKI Y AND WHEELOCK EF. (1987). Interferon-γ synergizes with tumor necrosis factor and with interleukin 1 and requires the presence of both monokines to induce cytotoxic activity in macrophages. *J. Immunol.*, **139**, 4096-4101.

CHOMCZYNSKI P AND SACCHI N. (1987). Single-step method of RNA isolation by acid guaniginium thiocyanate-phenol-chloroform extraction. *Anal. Biochem.*, **162**, 156-159.

COLEY WB. (1893). The treatment of malignant tumors by repeated inoculation of erysipelas: With a report of ten original cases. *Am. J. Med. Sci.*, **105**, 487-511.

DEMPSEY RA, DINARELLO CA, MIER JW, ROSENWASSER LJ, ALLEGRETTA M, BROWN TH AND PARKINSON DR. (1982). The differential effects of human leukocyte pyrogen/lymphocyte-activating factor, T cell growth factor, and interferon on human natural killer cell activity. *J. Immunol.*, **129**, 2504-2510.

EGGERMONT AMM, LIENARD D, SCHRAFFORDT KOOPS H, ROSEN-KAIMER F AND LEJEUNE FJ. (1993). Treatment of irresectable soft-tissue sarcomas of the limbs by isolation perfusion with high-dose TNF-α in combination with interferon-γ and melphalan. In *Tumor Necrosis Factor: Molecular and Cellular Biology and Clinical Relevance*, Fiers W and Buurman W (eds) pp. 239-243. Karger: Basle.

FEINBERG B, KURZROCK R, TALPAZ M, BLICK M, SAKS S AND GUTTERMAN JU. (1988). A phase-I trial of intravenously administered tumor necrosis factor alpha in cancer patients. *J. Clin. Oncol.*, **6**, 1328-1334.

FRANSEN L, RUYSSCHAERT MR, VAN DER HEYDEN J AND FIERS W. (1986). Recombinant human tumor necrosis factor: species specificity for a variety of human and murine transformed cell lines. *Cell. Immunol.*, **100**, 260-267.

GRAU GE, TACCHINI-COTTIER F, VESIN C, MILON G, LOU JN, PIGUET PF AND JUILLARD P. (1993). TNF-induced microvascular pathology: active role for platelets and importance of the LFA-1/ICAM-1 interaction. *Eur. Cytokine Netw.*, **4**, 415-419.

LASTER SM, WOOD JG AND GOODING LR. (1988). Tumor necrosis factor can induce both apoptotic and necrotic forms of cell lysis. *J. Immunol.*, **141**, 2629-2634.

LIENARD D, DELMOTTE JJ, RENARD N, EWALENKO P AND LEJEUNE F. (1992). High-dose tumor necrosis factor alpha in combination with interferon-gamma and melphalan in isolation perfusion of the limbs for melanoma and sarcoma. *J. Clin. Oncol.*, **10**, 52-60.

LIENARD D, EGGERMONT AMM, SCHRAFFORDT KOOPS H, KROON BBN, ROSENKAIMER F, AUTIER P AND LEJEUNE F. (1994). Isolated perfusion of the limb with high-dose TNF-α, IFNγ and melphalan for melanoma stage III: results of a multicentric pilot study. *Melanoma Res.*, **4** (suppl. 1), 21-26.

MULE JJ, McINTOSH JK, JABLONS DM AND ROSENBERG SA. (1990). Antitumor activity of recombinant interleukin-6 in mice. *J. Exp. Med.*, **171**, 629-636.

NAWROTH PP AND STERN DM. (1986). Modulation of endothelial cell hemostatic properties by tumor necrosis factor. *J. Exp. Med.*, **163**, 740-745.

NAYLOR MS, STAMP GWH, FOULKES WD, ECCLES D AND BALKWILL FR. (1993). Tumor Necrosis Factor and its receptors in human ovarian cancer - potential role in disease progession. *J. Clin. Invest.*, **91**, 2194-2206.

POBER JS, GIMBRONE JR MA, LAPIERRE, LA, MENDRICK DL, FIERS W, ROTHLEIN R AND SPRINGER TA. (1986). Overlapping patterns of activation of human endothelial cells by interleukin 1, tumor necrosis factor and immune interferon. *J. Immunol.*, **137**, 1893-1896.

PROIETTI E, BELARDELLI F, CARPINELLI G, DI VITO M, WOODROW D, MOSS J, SESTILI P, FIERS W, GRESSER I AND PODO F. (1988). Tumor necrosis factor α induces early morphologic changes and metabolic alterations in Friend leukemia cell tumors and fibrosarcomas in mice. *Int. J. Cancer*, **42**, 582-591.

RENARD N, LIENARD D, LESPAGNARD L, EGGERMONT A, HEIMANN R AND LEJEUNE F. (1994). Early endothelium activation and polymorphonuclear cell invasion precede specific necrosis of human melanoma and sarcoma treated by intravascular high-dose tumor necrosis factor alpha (rTNFα). *Int. J. Cancer*, **57**, 656-663.

ROBAYE B, MOSSELMANS R, FIERS W, DUMONT JE AND GALAND P. (1991). Tumor necrosis factor induces apoptosis (programmed cell death) in normal endothelial cells *in vitro*. *Am. J. Pathol.*, **138**, 447-453.

SHIMOMURA K, MANDA T, AND MUKUMOTO S. (1988). Recombinant tumor necrosis factor-alpha: Thrombus formation is the cause of anti-tumor activity. *Int. J. Cancer*, **41**, 243-247.

SRINIVASAN JM, FAJARDO LF AND HAHN GM. (1990). Mechanism of anti-tumor activity of tumor necrosis factor α with hyperthermia in a tumor necrosis factor α-resistant tumor. *J. Natl Cancer Inst.*, **82**, 1904-1910.

THOM AK, FRAKER DL, TAUBENBERGER JK AND NORTON JA. (1992). Effective regional therapy of experimental cancer with paralesional administration of tumor necrosis factor-α and interferon-γ. *Surg Oncol.*, **1**, 291-298.

THOM AK, ALEXANDER HR, ANDRICH MP, BARKER WC, ROSENBERG SA AND FRAKER DL. (1995). Cytokine levels and systemic toxicity in patients undergoing isolated limb perfusion with high-dose tumor necrosis factor, interferon gamma and melphalan. *J. Clin. Oncol.*, **13**, 264-273.

VAGLINI M, BELLI F, AMMATUNA M, INGLESE MG, MANZI R, PRADA A., PERSIANI L, SANTINAMI M, SANTORO N AND CASCINELLI N. (1994). Treatment of primary or relapsing limb cancer by isolation perfusion with high dose alpha-tumor necrosis factor, gamma-interferon and melphalan. *Cancer*, **73**, 483-492.

WATANABE N, NIITSU Y AND UMENO H. (1988). Toxic effects of tumor necrosis factor on tumor vasculature in mice. *Cancer Res.*, **48**, 2179-2183.


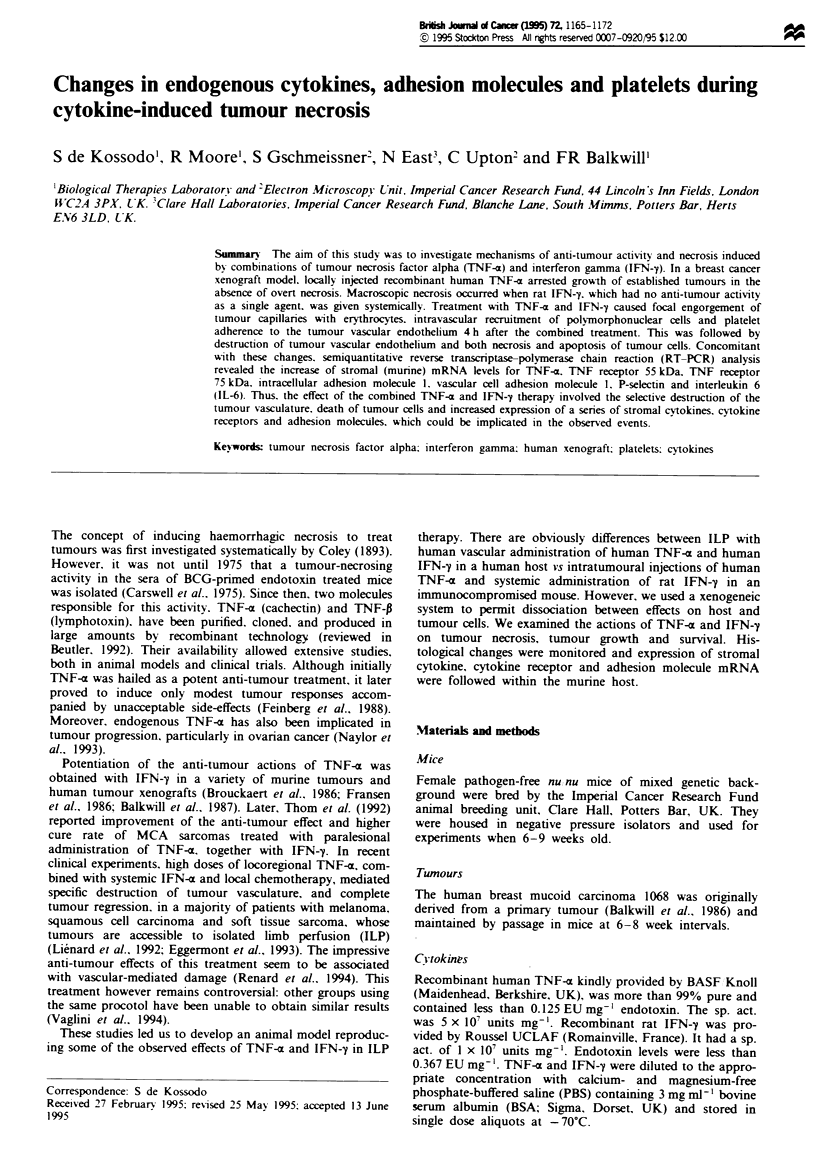

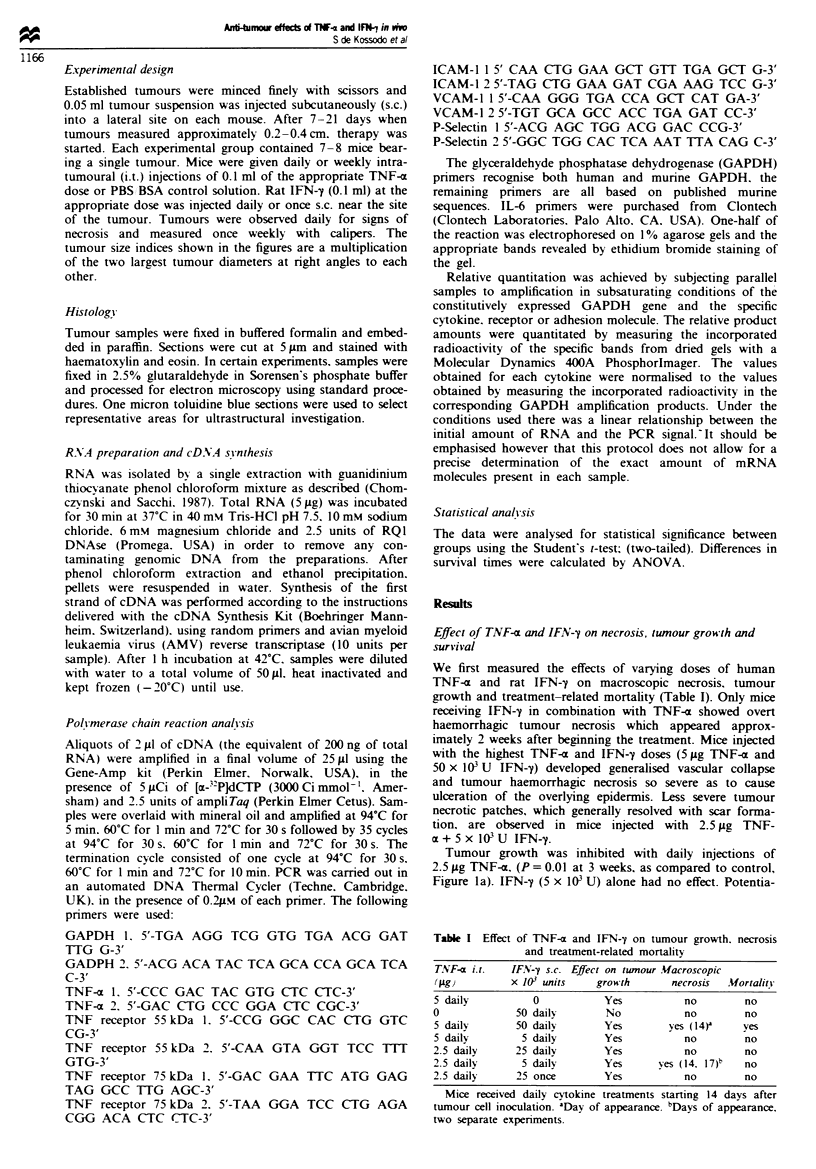

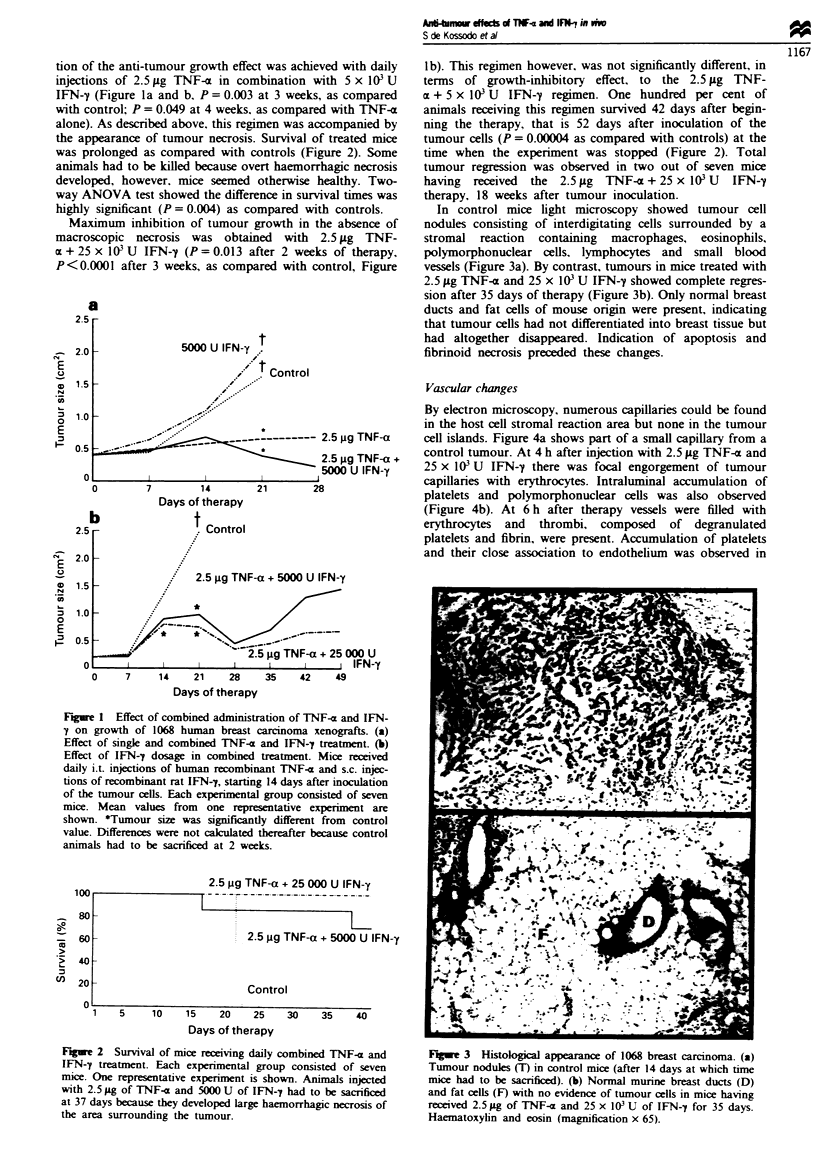

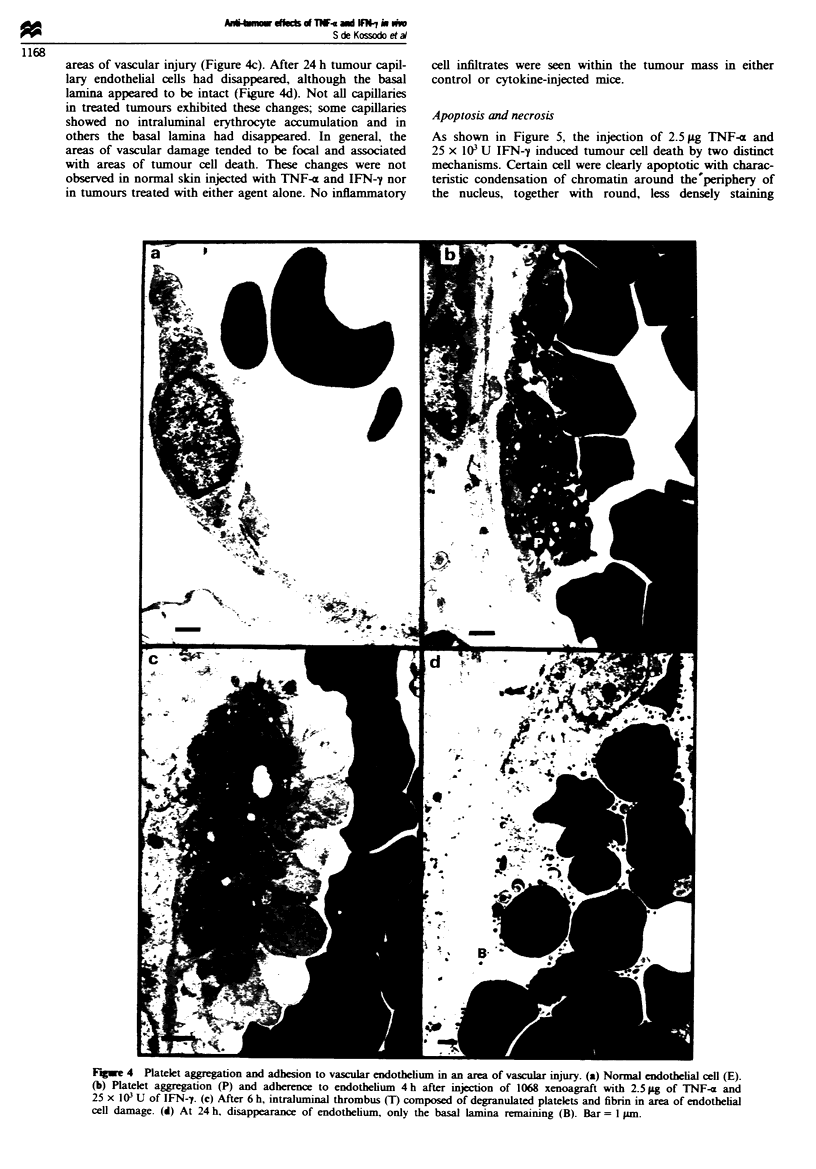

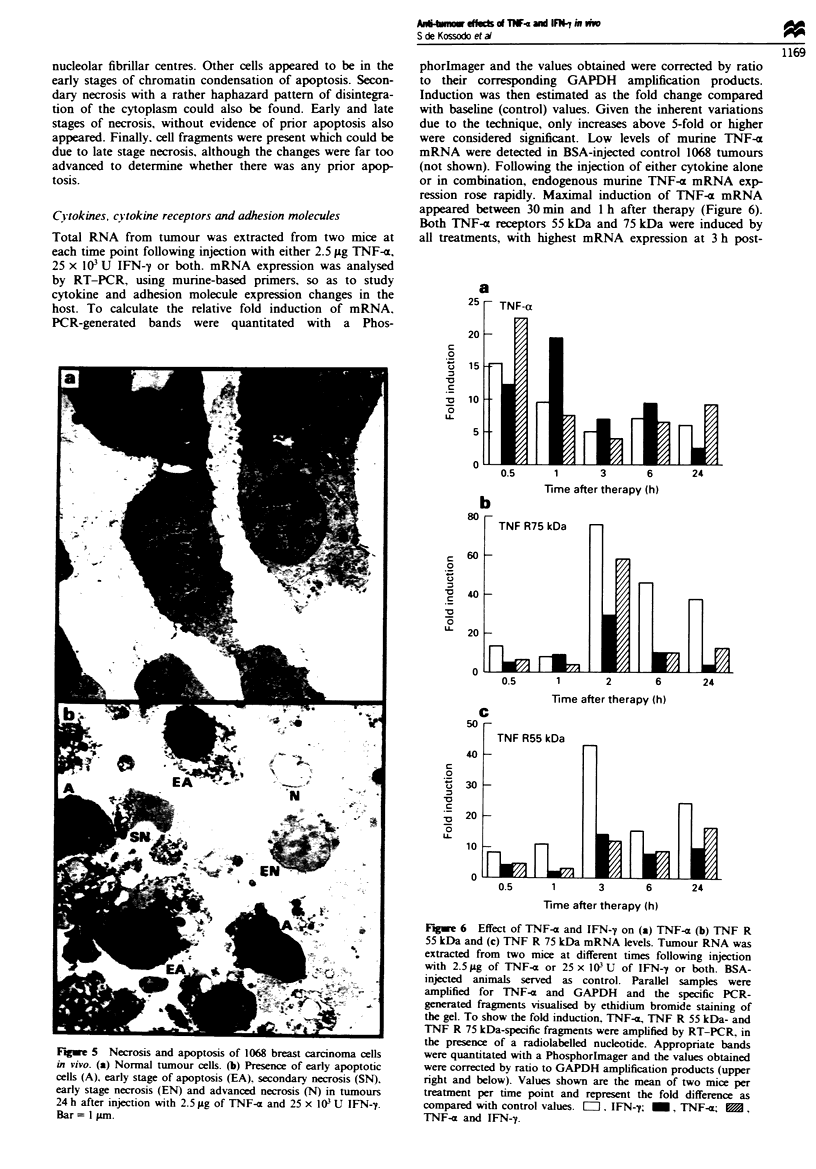

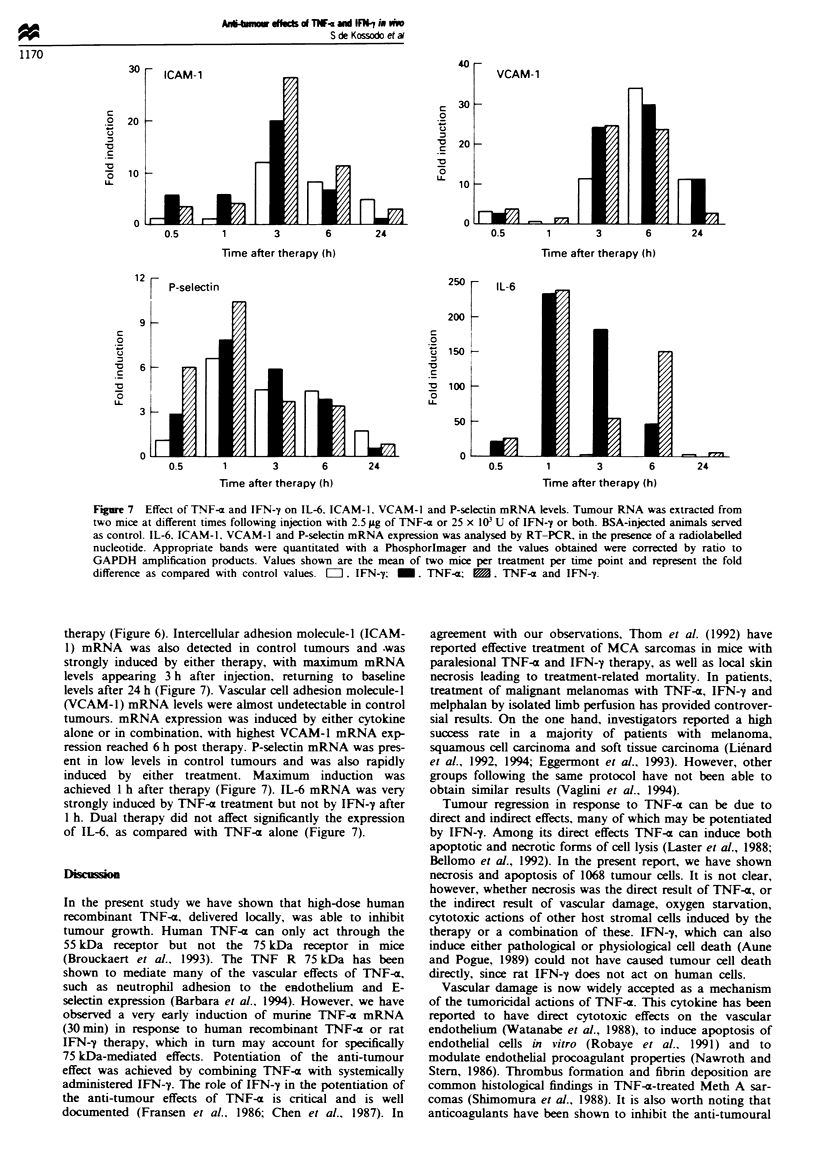

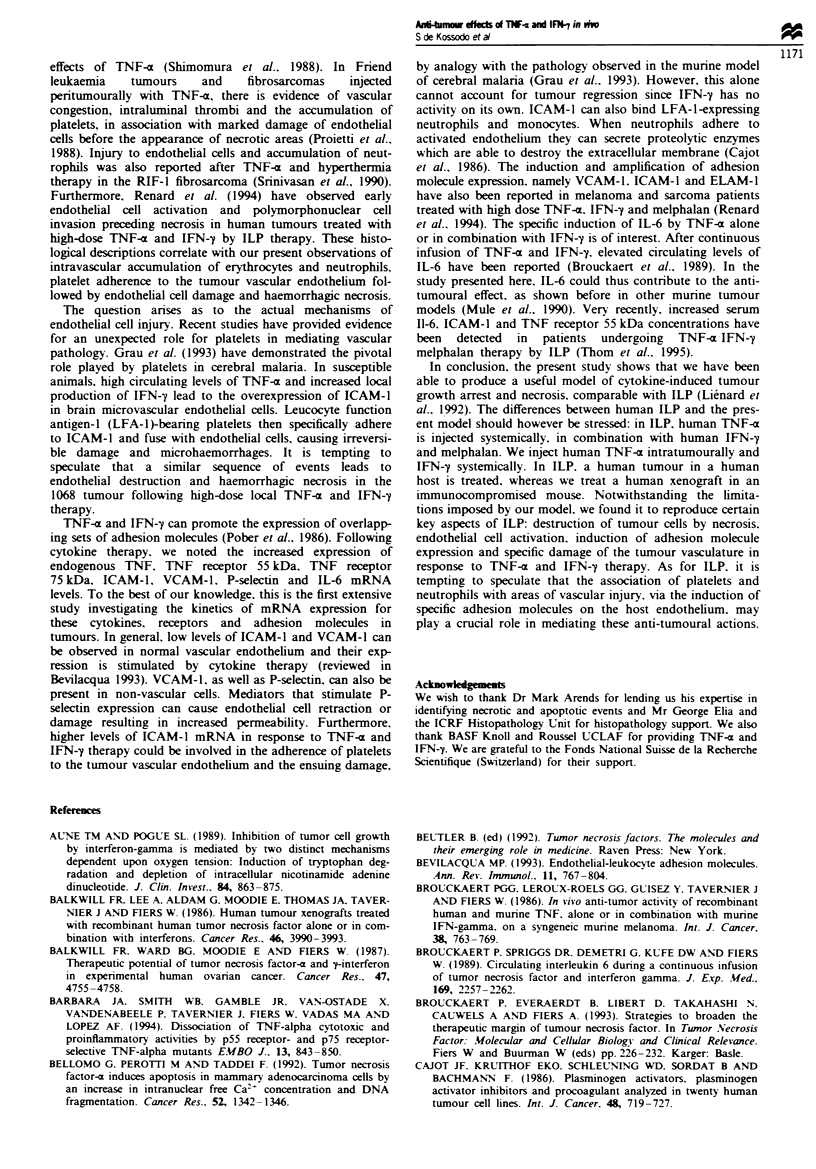

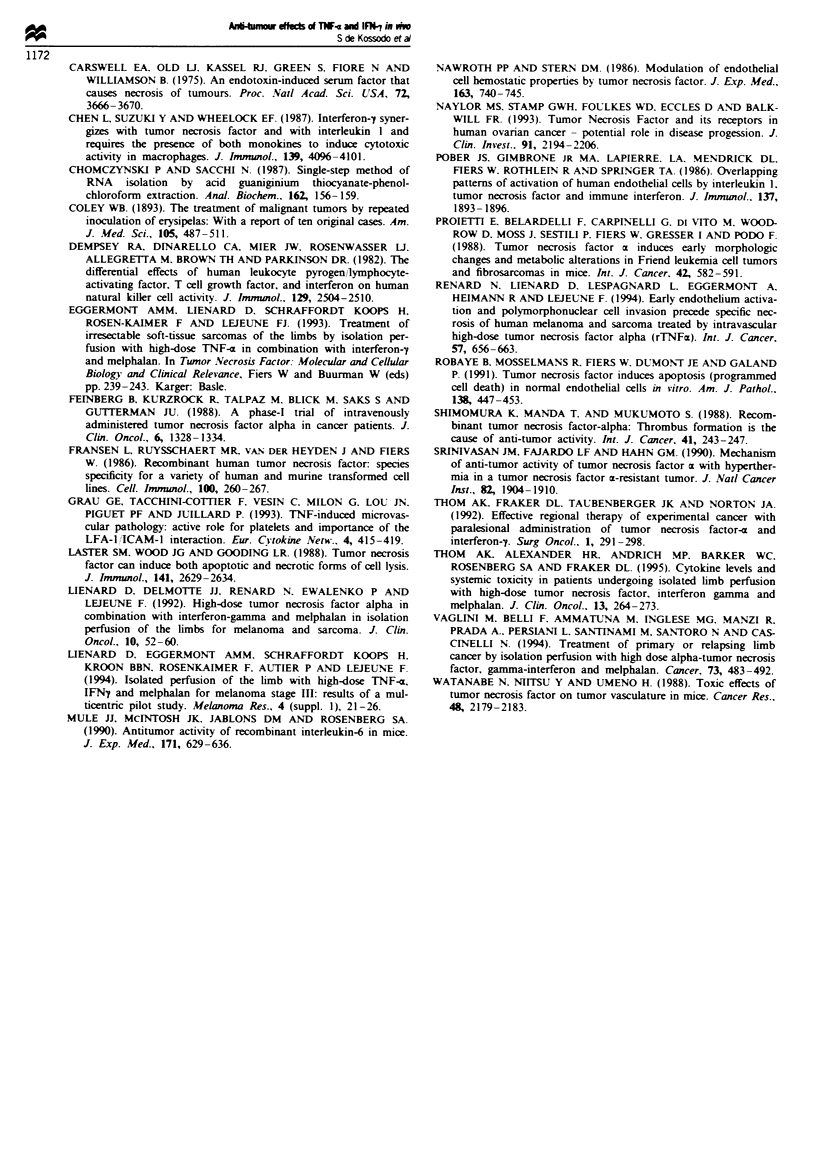


## References

[OCR_00984] Aune T. M., Pogue S. L. (1989). Inhibition of tumor cell growth by interferon-gamma is mediated by two distinct mechanisms dependent upon oxygen tension: induction of tryptophan degradation and depletion of intracellular nicotinamide adenine dinucleotide.. J Clin Invest.

[OCR_00992] Balkwill F. R., Lee A., Aldam G., Moodie E., Thomas J. A., Tavernier J., Fiers W. (1986). Human tumor xenografts treated with recombinant human tumor necrosis factor alone or in combination with interferons.. Cancer Res.

[OCR_00997] Balkwill F. R., Ward B. G., Moodie E., Fiers W. (1987). Therapeutic potential of tumor necrosis factor-alpha and gamma-interferon in experimental human ovarian cancer.. Cancer Res.

[OCR_01004] Barbara J. A., Smith W. B., Gamble J. R., Van Ostade X., Vandenabeele P., Tavernier J., Fiers W., Vadas M. A., Lopez A. F. (1994). Dissociation of TNF-alpha cytotoxic and proinflammatory activities by p55 receptor- and p75 receptor-selective TNF-alpha mutants.. EMBO J.

[OCR_01008] Bellomo G., Perotti M., Taddei F., Mirabelli F., Finardi G., Nicotera P., Orrenius S. (1992). Tumor necrosis factor alpha induces apoptosis in mammary adenocarcinoma cells by an increase in intranuclear free Ca2+ concentration and DNA fragmentation.. Cancer Res.

[OCR_01020] Bevilacqua M. P. (1993). Endothelial-leukocyte adhesion molecules.. Annu Rev Immunol.

[OCR_01025] Brouckaert P. G., Leroux-Roels G. G., Guisez Y., Tavernier J., Fiers W. (1986). In vivo anti-tumour activity of recombinant human and murine TNF, alone and in combination with murine IFN-gamma, on a syngeneic murine melanoma.. Int J Cancer.

[OCR_01029] Brouckaert P., Spriggs D. R., Demetri G., Kufe D. W., Fiers W. (1989). Circulating interleukin 6 during a continuous infusion of tumor necrosis factor and interferon gamma.. J Exp Med.

[OCR_01044] Cajot J. F., Kruithof E. K., Schleuning W. D., Sordat B., Bachmann F. (1986). Plasminogen activators, plasminogen activator inhibitors and procoagulant analyzed in twenty human tumor cell lines.. Int J Cancer.

[OCR_01056] Carswell E. A., Old L. J., Kassel R. L., Green S., Fiore N., Williamson B. (1975). An endotoxin-induced serum factor that causes necrosis of tumors.. Proc Natl Acad Sci U S A.

[OCR_01059] Chen L., Suzuki Y., Wheelock E. F. (1987). Interferon-gamma synergizes with tumor necrosis factor and with interleukin 1 and requires the presence of both monokines to induce antitumor cytotoxic activity in macrophages.. J Immunol.

[OCR_01067] Chomczynski P., Sacchi N. (1987). Single-step method of RNA isolation by acid guanidinium thiocyanate-phenol-chloroform extraction.. Anal Biochem.

[OCR_01075] Dempsey R. A., Dinarello C. A., Mier J. W., Rosenwasser L. J., Allegretta M., Brown T. E., Parkinson D. R. (1982). The differential effects of human leukocyte pyrogen/lymphocyte-activating factor, T cell growth factor, and interferon on human natural killer activity.. J Immunol.

[OCR_01093] Feinberg B., Kurzrock R., Talpaz M., Blick M., Saks S., Gutterman J. U. (1988). A phase I trial of intravenously-administered recombinant tumor necrosis factor-alpha in cancer patients.. J Clin Oncol.

[OCR_01099] Fransen L., Ruysschaert M. R., Van der Heyden J., Fiers W. (1986). Recombinant tumor necrosis factor: species specificity for a variety of human and murine transformed cell lines.. Cell Immunol.

[OCR_01105] Grau G. E., Tacchini-Cottier F., Vesin C., Milon G., Lou J. N., Piguet P. F., Juillard P. (1993). TNF-induced microvascular pathology: active role for platelets and importance of the LFA-1/ICAM-1 interaction.. Eur Cytokine Netw.

[OCR_01111] Laster S. M., Wood J. G., Gooding L. R. (1988). Tumor necrosis factor can induce both apoptic and necrotic forms of cell lysis.. J Immunol.

[OCR_01114] Lienard D., Ewalenko P., Delmotte J. J., Renard N., Lejeune F. J. (1992). High-dose recombinant tumor necrosis factor alpha in combination with interferon gamma and melphalan in isolation perfusion of the limbs for melanoma and sarcoma.. J Clin Oncol.

[OCR_01124] Liénard D., Eggermont A. M., Schraffordt Koops H., Kroon B. B., Rosenkaimer F., Autier P., Lejeune F. J. (1994). Isolated perfusion of the limb with high-dose tumour necrosis factor-alpha (TNF-alpha), interferon-gamma (IFN-gamma) and melphalan for melanoma stage III. Results of a multi-centre pilot study.. Melanoma Res.

[OCR_01128] Mulé J. J., McIntosh J. K., Jablons D. M., Rosenberg S. A. (1990). Antitumor activity of recombinant interleukin 6 in mice.. J Exp Med.

[OCR_01135] Nawroth P. P., Stern D. M. (1986). Modulation of endothelial cell hemostatic properties by tumor necrosis factor.. J Exp Med.

[OCR_01141] Naylor M. S., Stamp G. W., Foulkes W. D., Eccles D., Balkwill F. R. (1993). Tumor necrosis factor and its receptors in human ovarian cancer. Potential role in disease progression.. J Clin Invest.

[OCR_01144] Pober J. S., Gimbrone M. A., Lapierre L. A., Mendrick D. L., Fiers W., Rothlein R., Springer T. A. (1986). Overlapping patterns of activation of human endothelial cells by interleukin 1, tumor necrosis factor, and immune interferon.. J Immunol.

[OCR_01154] Proietti E., Belardelli F., Carpinelli G., Di Vito M., Woodrow D., Moss J., Sestili P., Fiers W., Gresser I., Podo F. (1988). Tumor necrosis factor alpha induces early morphologic and metabolic alterations in Friend leukemia cell tumors and fibrosarcomas in mice.. Int J Cancer.

[OCR_01158] Renard N., Liénard D., Lespagnard L., Eggermont A., Heimann R., Lejeune F. (1994). Early endothelium activation and polymorphonuclear cell invasion precede specific necrosis of human melanoma and sarcoma treated by intravascular high-dose tumour necrosis factor alpha (rTNF alpha).. Int J Cancer.

[OCR_01168] Robaye B., Mosselmans R., Fiers W., Dumont J. E., Galand P. (1991). Tumor necrosis factor induces apoptosis (programmed cell death) in normal endothelial cells in vitro.. Am J Pathol.

[OCR_01174] Shimomura K., Manda T., Mukumoto S., Kobayashi K., Nakano K., Mori J. (1988). Recombinant human tumor necrosis factor-alpha: thrombus formation is a cause of anti-tumor activity.. Int J Cancer.

[OCR_01179] Srinivasan J. M., Fajardo L. F., Hahn G. M. (1990). Mechanism of antitumor activity of tumor necrosis factor alpha with hyperthermia in a tumor necrosis factor alpha-resistant tumor.. J Natl Cancer Inst.

[OCR_01191] Thom A. K., Alexander H. R., Andrich M. P., Barker W. C., Rosenberg S. A., Fraker D. L. (1995). Cytokine levels and systemic toxicity in patients undergoing isolated limb perfusion with high-dose tumor necrosis factor, interferon gamma, and melphalan.. J Clin Oncol.

[OCR_01185] Thom A. K., Fraker D. L., Taubenberger J. K., Norton J. A. (1992). Effective regional therapy of experimental cancer with paralesional administration of tumour necrosis factor-alpha + interferon-gamma.. Surg Oncol.

[OCR_01196] Vaglini M., Belli F., Ammatuna M., Inglese M. G., Manzi R., Prada A., Persiani L., Santinami M., Santoro N., Cascinelli N. (1994). Treatment of primary or relapsing limb cancer by isolation perfusion with high-dose alpha-tumor necrosis factor, gamma-interferon, and melphalan.. Cancer.

[OCR_01202] Watanabe N., Niitsu Y., Umeno H., Kuriyama H., Neda H., Yamauchi N., Maeda M., Urushizaki I. (1988). Toxic effect of tumor necrosis factor on tumor vasculature in mice.. Cancer Res.

